# Editorial: Clinical management of older persons with sarcopenia: current status and future directions

**DOI:** 10.3389/fmed.2024.1496036

**Published:** 2024-09-27

**Authors:** Paolo Gallo

**Affiliations:** Department of Medicine and Surgery, Campus Bio-Medico University Hospital, Rome, Italy

**Keywords:** sarcopenia, muscle strength, handgrip, β-hydroxy-β-methylbutyrate (HMB), ultrasound

Sarcopenia has been recognized as a systemic disorder strictly connected with patients' health and outcomes. Characterized by the loss of muscle strength, mass, and physical performance, sarcopenia poses a serious threat to the wellbeing of the aging population, leading to increased risk of falls, frailty and a range of chronic diseases. Recent research highlights the need for increased awareness, better diagnostic criteria and effective intervention strategies to mitigate the impact of this debilitating condition. Some studies have pointed out the significant impact of nutritional status on sarcopenia, particularly in relation to the risk of malnutrition. In this Research Topic, we have focused on manuscripts that provide new insights into the case finding of sarcopenic patients, advances in biomarkers and molecular mechanisms underlying this chronic disease, and into the role of early diagnostic and therapeutic intervention.

Vidaña-Espinoza et al.'s most recent research focuses on the relationship between malnutrition and sarcopenia, particularly in older adults. Cross-sectional studies and systematic reviews have consistently found that older adults at risk of malnutrition, as diagnosed by tools such as the Mini-Nutritional Assessment (MNA), are more likely to develop sarcopenia. The study also found that people at risk of malnutrition, as diagnosed by the MNA, were almost twice as likely to develop sarcopenia over time. However, this association lost statistical significance after adjustment for various covariates, suggesting that other factors, such as body fat, may mediate the relationship between malnutrition and sarcopenia. Overall, the high risk of malnutrition in this Mexican population highlights the need for proactive measures to address malnutrition as a key factor in the prevention of sarcopenia. Indeed, the interaction between malnutrition and sarcopenia is relevant, as both conditions can exacerbate each other, leading to a downward spiral of physical decline and increased vulnerability to other health problems.

Ginevičiene et al. review recent advances in the physiopathology of sarcopenia and new potential biomarkers. The search for reliable biomarkers is crucial for early diagnosis and development of targeted therapies. Recent studies have identified inflammatory cytokines and myokines involved in muscle degradation as good candidates. Proteomic and genetic markers are also being explored and offer promising avenues for understanding the molecular mechanisms underlying the disease. In the future, a combination of multiple stratification of genetic biomarkers may be required to more accurately predict frailty and sarcopenia and to evaluate the outcomes of related interventions.

A simple, inexpensive and useful tool for screening and diagnosis is key to patient management and improved outcomes. Prell et al. highlight the use of muscle ultrasound as a valuable tool for assessing muscle mass and structure. This method allows early detection of muscle wasting, allowing healthcare providers to intervene in a timely manner. Ultrasound can measure multiple muscle parameters such as thickness, cross-sectional area and echogenicity, providing a comprehensive assessment of muscle health. By identifying sarcopenia early, healthcare providers can implement strategies to slow its progression and improve the quality of life of older adults.

Finally, we chose a systematic review and meta-analysis of one of the most debated dietary supplements, which has gained attention as a potential intervention in the medical management of sarcopenia. Su et al. conducted this review on β-hydroxy-β-methylbutyrate (HMB), a metabolite of the amino acid leucine, which has shown promise in improving muscle protein turnover, making it a potential candidate for the treatment of sarcopenia. The results showed a significant improvement in handgrip strength, a key indicator of muscle function, in those supplemented with HMB. However, the evidence for its effects on other parameters, such as muscle mass or gait speed, remains inconclusive and its role in the comprehensive management of sarcopenia needs to be further investigated.

In conclusion, sarcopenia is a growing public health concern with significant implications for the aging population worldwide. Its multifactorial nature, including aging, chronic diseases and malnutrition, requires a comprehensive approach to diagnosis, prevention and treatment. The development of reliable biomarkers and the use of advanced diagnostic tools such as muscle ultrasound are essential for early detection and intervention. Nutritional supplementation, particularly with HMB, is a promising treatment option, although further research is needed to fully understand its potential. As the world's population continues to age, the burden of sarcopenia is likely to increase, making it imperative that healthcare systems prioritize research and policy development in this area.

